# The comparative analysis of statistics, based on the likelihood ratio criterion, in the automated annotation problem

**DOI:** 10.1186/1471-2105-9-31

**Published:** 2008-01-22

**Authors:** Andrey M Leontovich, Konstantin Y Tokmachev, Hans C van Houwelingen

**Affiliations:** 1 Belozersky Institute of Physico-Chemical Biology, Moscow State University, Moscow, 119899, Russia; 2 Department of Medical Statistics and Bioinformatics, Leiden University Medical Center, Post zone S5-P, 2300 RC Leiden, The Netherlands

## Abstract

**Background:**

This paper discusses the problem of automated annotation. It is a continuation of the previous work on the A^4^-algorithm (Adaptive algorithm of automated annotation) developed by Leontovich and others.

**Results:**

A number of new statistics for the automated annotation of biological sequences is introduced. All these statistics are based on the likelihood ratio criterion.

**Conclusion:**

Some of the statistics yield a prediction quality that is significantly higher (up to 1.5 times higher) in comparison with the results obtained with the A^4^-procedure.

## Background

Many biological databanks, both dealing with protein sequences (e.g., SWISS-PROT) and nucleotide sequences (e.g., GeneBank), contain not only primary structures of sequences (i.e., sequences of letters – amino acids or nucleotides), but also information about functions and properties of these sequences. This information is stored in so called *description fields *of the sequences. There exist different types of description fields – KW (KeyWords), DE (Descriptions), ..., FT (Feature Table), ...; elements of description fields are referred to as *words*. Words from KW, DE, ... fields describe a sequence as a whole, while words from FT fields correspond to certain positions (letters) of a sequence.

The automated annotation problem can be described as follows. Consider a biological sequence (referred to as a query sequence) with known primary structure (i.e. letter sequence) but unknown properties and functions (i.e., description fields). The task is to determine functions and properties of this sequence (in other words, to restore its description fields) on the basis of the primary structure. The annotation should be fully automated. This is the subject of the current paper.

There are two main approaches to the solution of this problem. In the first approach (it can be called a *static *one) a certain fixed protein classification (grouping proteins according to similarity in structure and/or functions), specified beforehand, is used: for a query protein the search of a relative group (super family) is performed on the basis of primary structures, and properties/functions of this group are extended to the query protein. An example of this approach is described in the paper by W. Fleischmann et al. [1], it uses the protein classification (more than 1000 families), stored in the Prosite databank.

The second approach (it can be called a *dynamic *or an *adaptive *one) does not use protein classification. Instead, a "dynamic" collection of bank sequences that are similar to a query sequence is generated, and then common properties/functions of these bank sequences are extended to the query sequence. One of the first examples of this approach was described by M.A. Andrade et al. [2]. In this paper the prediction was based on so called *word reliability function *– a function depending on the degree of similarity between a query sequence and corresponding bank sequences. In other examples of annotation procedures based on the dynamic approach prediction was performed in a "naive" way – all properties/functions of similar proteins were extended to a query protein, or using stochastic methods – only properties/functions that are most frequent for the collection of similar proteins were extended (see [3,4]).

The current paper uses the dynamic approach. This paper is a sequel to paper [5] that describes the *A*^4 ^algorithm (the Adaptive Algorithm of Automated Annotation), so results of the paper [5] are constantly used here. The *A*^4 ^algorithm is based on a stochastic approach. More precisely, it is based on the notion of *transfer probabilities*. Transfer probabilities are the probabilities of word transfer (extension) from description fields of one sequence to description fields of another sequence; they depend on the measure of similarity between sequences. Transfer probabilities are evaluated on the basis of word transfer frequencies in the found collection of sequences similar to a query sequence. For each word from description fields of sequences included in the collection the prediction of the fact that this word belongs (or does not belong) to the description field of a query sequence is performed; this prediction is based on transfer probabilities.

In the current paper we introduce and analyze a number of new statistics for the prediction. All of them are based on the likelihood ratio criterion [6] (which is the most powerful criterion). As in [5], all these statistics are evaluated using transfer probabilities. Two approaches to statistics definition are introduced: a "discrete" approach and a "continuous" approach. A detailed analysis and comparison of introduced statistics are performed and the best statistics are selected.

The emphasis is on a precise description of the way these statistics can be constructed using well-known concepts from statistical decision theory.

The current *A*^4 ^algorithm uses SPKW as a language of annotation. Of course, it is possible to use GO terms in *A*^4 ^as well. That would facilitate comparison with other approaches. However, at the current stage of our research we test and choose "the best decision making algorithm", not "the best annotation terminology". Obviously, which annotation is used hardly matters for the problem of finding the optimal "decision making algorithm"

## Results

### Algorithm description

#### Generation of a collection of similar sequences

First, we introduce some notation. For the sake of brevity we write "a word *ω *belongs to a sequence *π*" instead of "a word *ω *belongs to description fields of a sequence *π*". If *ω *is a KW-type word (to be definite, further in the paper we consider only amino-acid sequences and KW-type words), we write *ω *∈ *KW*[*π*].

The application of an *annotation *procedure to an unannotated amino-acid query sequence (i.e., the prediction of description fields) starts with generating a collection of sequences *similar *to this query sequence with known description fields. These similar sequences are selected from a certain databank that contains annotated amino-acid sequences, e.g., SWISS-PROT.

There exist different approaches to the generation of a collection of similar sequences (see [7]). This collections can be generated on the basis of global alignments between a query sequence and bank sequences (global alignments can be constructed, e.g., by CLUSTAL procedure) using *an identity percentage *or, more generally, a *similarity percentage *as a similarity measure. Another variant is to use local alignments (i.e., alignments of most similar fragments of compared sequences, see [7]) instead of global alignments. Local alignments can be constructed, for example, by a well-known BLAST procedure [7,8], in which sum of weights or a corresponding e-value serves as a measure of similarity between fragments (and thus between compared sequences). Other alignment procedures are also acceptable; alignment procedure selection does not play a critical role.

Since we build on the *A*^4 ^procedure, we briefly summarize that approach. In the *A*^4 ^procedure a collection of similar sequences is generated on the basis of local alignments of a special type. These alignments are constructed by the DotHelix procedure ([9]), in which the "*power" *(sum of weights divided by the root of the length of the local alignment, see [9,10] for details) serves as a similarity measure. Each local alignment constructed by DotHelix procedure is a chain of *closely located gapless local alignments*. Local alignments that are generated during the construction of a collection of similar sequences are referred to as *primary *local alignments.

Each sequence from a collection of similar sequences *π*_1_,...,*π*_*n *_can have several corresponding primary local alignments, but for the sake of simplicity we assume that each similar sequence *π*_*i *_has exactly one corresponding primary local alignment, the one with the maximum similarity measure. Let *μ*_*i *_denote the similarity measure (power) of a primary alignment that corresponds to *π*_*i*_. The value of *μ*_*i *_characterizes the measure of similarity between fragments that constitute this alignment; at the same time *μ*_*i *_can be treated as a measure of similarity between the whole query sequence *π*_0 _and the whole similar sequence *π*_*i*_. We assume that similar sequences are ordered in such a way that *μ*_*1 *_≥ *μ*_2 _≥...≥ *μ*_*n*_.

#### The exact stochastic formulation of the problem

Let *π*_0 _be an unannotated amino-acid query sequence, *π*_1_,...,*π*_*n *_be a collection of sequences similar to *π*_0_, and *ω *be a word that belongs to some similar sequences (i.e., *ω *∈ *KW*[*π*_*i*_]. for at least one similar sequence *π*_*i*_). The task is to predict whether this word *ω *belongs to the query sequence *π*_0 _or not.

Let us put

*ξ*_*i *_= 1, if *ω *∈ *KW*[*π*_*i*_], and *ξ*_*i *_= 0, if *ω *∉ *KW*[*π*_*i*_]   (*i *= 1,...,*n*).

we also put

*ξ*_0 _= 1, if *ω *∈ *KW*[*π*_0_], and *ξ*_0 _= 0, if *ω *∉ *KW*[*π*_0_].

Variables *ξ*_*i *_can be treated as random variates Actually, they depend on *ω*. For the sake of brevity we write *ξ*_*i *_instead of *ξ*_*i*_(*ω*).

In this notation the problem can be stated as follows. Measures of similarity *μ*_*i *_between the query sequence *π*_0 _and similar sequences *π*_*i *_and values of random variates *ξ*_*i*_, *i *= 1,...,*n*, are given. The task is to determine whether the word *ω *belongs to the query sequence *π*_0 _or not. In other words, two hypotheses are considered, *H*_1 _: *ξ*_0 _= 1 (i.e., *ω *∈ *KW*[*π*_0_]), and *H*_0 _: *ξ*_0 _= 0 (i.e., *ω *∉ *KW*[*π*_0_]), and the task is to construct a procedure that allows to decide which hypothesis is true on the basis of *ξ *= (*ξ*_1_,...,*ξ*_*n*_). As announced in the introduction, we base our procedures on the likelihood ratio. Let us recall the famous Bayes' Theorem that can be written as

$$\frac{P\{H_{1}|\xi \}}{P\{H_{0}|\xi \}}=\frac{\text{P}\{\xi_{0}=1|\xi \}}{\text{P}\{\xi_{0}=0|\xi \}}=\frac{\text{P}\{\xi_{0}=1,\xi \}/\text{P}\{\xi \}}{\text{P}\{\xi_{0}=0,\xi \}/\text{P}\{\xi \}}=\frac{\text{P}\{\xi |\xi_{0}=1\}}{\text{P}\{\xi |\xi_{0}=0\}}\cdot \frac{\text{P}\{\xi_{0}=1\}}{\text{P}\{\xi_{0}=0\}}.$$

Here the left part is the *posterior odds*, that is the ratio of *a posteriori *probabilities of hypotheses *H*_1 _and *H*_0 _(a posteriori means that values *ξ*_1_,...,*ξ*_*n *_are known). It is equal to the product of the likelihood ratio $\frac{\text{P}\{\xi |\xi_{0}=1\}}{\text{P}\{\xi |\xi_{0}=0\}}$ (i.e., the ratio of probabilities that the set of values *ξ *= *ξ*(*ω*) is realized for the word *ω *given the conditions *ξ*_0 _= 1 and *ξ*_0 _= 0 respectively) and the *prior odds*, that is the ratio of a priori probabilities of hypotheses *H*_1 _and *H*_0_.

Statistical decision theory tells us that the optimal prediction procedure should be based on the statistic $\frac{\text{P}\{H_{1}|\xi \}}{\text{P}\{H_{0}|\xi \}}$ or equivalently on the likelihood ratio. For any statistic a threshold value should be specified for the procedure: if the value of the statistic is greater than the threshold, hypothesis *H*_1 _is accepted, otherwise hypothesis *H*_0 _is accepted. The threshold value should be selected in such a way that the total number of incorrect predictions (i.e., the sum of the number of type 1 errors and the number of type 2 errors) would be minimal. It is clear that if the prior odds are equal to 1, then a threshold value of one should be selected for the likelihood ratio; total number of errors would (theoretically) be minimal for this threshold. Surely, the assumption that the ratio of a priori hypothesis probabilities equals 1 does not seem to be natural. Indeed, the number of considered words that do not belong to a query sequence is much greater (approximately 8 times greater) than the number of considered words that belong to a query sequence. But statistics that are obtained from the assumption that this a priori ratio equals 1, and the assumption that this a priori ratio does not equal 1, but is *constant *(i.e., it does not depend on a word *ω*) are equivalent. Essentially these are the same statistics (only the threshold value should be changed: a value $\frac{\text{P}\{\xi_{0}=0\}}{\text{P}\{\xi_{0}=1\}}$ should be taken instead of 1; as it was noted, this value approximately equals 8 in our data). Therefore, we assume from now on that the ratio of a priori hypothesis probabilities equals 1. Thus all considered statistics are based on the likelihood ratio

(1) $$\frac{\text{P}\{\xi |\xi_{0}=1\}}{\text{P}\{\xi |\xi_{0}=0\}}$$

#### Assumption of independence of variables *ξ*_*i*_. Transfer probabilities

By virtue of equation (1) we need to estimate conditional probabilities P{*ξ*|*ξ*_0 _= *ε*}, where *ε *= 1 or 0 in order to calculate the likelihood ratio. Our derivation of these estimates uses the assumption that variables *ξ*_*i*_, *i *= 1,...,*n*, are independent in the aggregate. Surely, this assumption is false. In reality variables *ξ*_*i *_are dependent, and the dependence is considerably strong. Nevertheless, in our definition of the likelihood ratio statistic (and the statistic that is the logarithm of the likelihood ratio) we use the independence assumption. Since variables *ξ*_*i *_are not independent, one can not assert that the obtained statistics are the most powerful, but these statistics can be still quite good. In decision theory, this approach is known as the naive Bayes procedure.

The independence of variables *ξ*_*i *_implies the equality

(2) $$\begin{array}{cc} \text{P}\{\xi |\xi_{0}=\varepsilon \}=\prod_{i=1}^{n}\text{P}\{\xi_{i}|\xi_{0}=\varepsilon \}, & \varepsilon =1,0 \end{array}$$

Each variable *ξ*_*i *_has exactly two possible values: 1 and 0. Thus, everything is reduced to the following four conditional probabilities:

(3) P{*ξ*_*i *_= 1|*ξ*_0 _= 1},   P{*ξ*_*i *_= 0|*ξ*_0_= 1},   P{*ξ*_*i *_= 1|*ξ*_0 _= 0},   P{*ξ*_*i *_= 0|*ξ*_0 _= 0}.

In addition, it is clear that

(4) $$\begin{array}{l} \text{P}\{\xi_{i}=1|\xi_{0}=1\}+\text{P}\{\xi_{i}=0|\xi_{0}=1\}=1,\\ \text{P}\{\xi_{i}=1|\xi_{0}=0\}+\text{P}\{\xi_{i}=0|\xi_{0}=0\}=1, \end{array}$$

so actually everything is reduced to two conditional probabilities

P{*ξ*_*i *_= 1|*ξ*_0 _= 1},   P{*ξ*_*i *_= 1|*ξ*_0_= 0}.

Conditional probabilities (3) are a special case of conditional probabilities of the type

P{*ξ*_*i *_= *ε*_1_|*ξ*_*j *_= *ε*_2_}, where *ε*_1_, *ε*_2 _= 1 or 0, *i*, *j *= 0,1,...,*n*

(above special case corresponds to j = 0). We call all this conditional probabilities *transfer probabilities *and denote them by

$$\text{P}\{\xi_{i}=\varepsilon_{1}|\xi_{j}=\varepsilon_{2}\}=p_{\varepsilon_{1}|\varepsilon_{2}}.$$

Transfer probabilities depend on i, j (and certainly on the word *ω*):

$$p_{\varepsilon_{1}|\varepsilon_{2}}=p_{\varepsilon_{1}|\varepsilon_{2}}(i,j;\omega ).$$

Conditional probabilities play a central role in our procedure. According to equation (4), it suffices to explain how transfer probabilities *p*_1|1_, *p*_1|0 _are evaluated.

We suppose that transfer probabilities satisfy the following assumptions ("axioms") (the sense of these assumptions is obvious).

*Assumption 1) *For a fixed word *ω *and for sequences *π*_0_, *π*_1_,...,*π*_*n *_(i.e., for the sequence *π*_0 _and sequences similar to *π*_0_) transfer probabilities depend only on the measure of similarity between sequences. Thus, we have:

*p*_1|1 _= *p*_1|1 _(*i*, *j*;*ω*) = *p*_1|1_(*μ*_*ij*_;*ω*) = *p*_1|1_(*μ*_*ij*_) = 1 - *p*_0|1_(*μ*_*ij*_),

*p*_1|0 _= *p*_1|0 _(*i*, *j*;*ω*) = *p*_1|0_(*μ*_*ij*_;*ω*) = *p*_1|0_(*μ*_*ij*_) = 1 - *p*_0|0_(*μ*_*ij*_),

where *μ*_*ij *_is the measure of similarity between sequences *π*_*i*_, *π*_*j*_(*i*, *j *= 0,1,...,*n*).

Particularly, if one of these sequences is the query sequence *π*_0 _and *μ*_*j *_is the measure of similarity between *π*_*j*_, *π*_0_, we have

(5) P{*ξ*_*j *_= 1|*ξ*_0 _= 1} = *p*_1|1_(*μ*_*j*_), P{*ξ*_*j *_= 1|*ξ*_0 _= 0} = *p*_1|0_(*μ*_*j*_).

*Assumption 2*) Transfer probabilities (for an arbitrary fixed word ω) depend on similarity measure *μ *monotonically: the probability *p*_1|1_(*μ*) increases (does not decrease) and the probability *p*_1|0_(*μ*)decreases (does not increase) as *μ *increases.

*Assumption 3*) The inequality

(6) *p*_1|1_(*μ*) > *p*_1|0_(*μ*)

always holds (if *μ *> 0).

Transfer probabilities are evaluated on the basis of the measure of similarity between similar sequences for which it is known whether *ω *∈ *KW*[*π*_*i*_] using the so called *isotonic regression *procedure (see [11]). (In [5] this procedure was referred to as *monotonization *procedure). Results of this procedure are piecewise-constant monotonous functions of the similarity measure *μ *that increase (do not decrease) for probabilities *p*_1|1_(*μ*) and decrease (do not increase) for probabilities *p*_1|0_(*μ*).

We briefly recall the isotonic regression problem. Let one have two number sets (i.e., a set of points in the plain) *x*_*i*_, *y*_*i*_, *i *= 1,...,*n*, for which *x*_1 _≤ *x*_2 _≤...≤ *x*_*n*_. The task is to find values *z*_1_, *z*_2_,...,*z*_*n*_, *z*_1 _≤ *z*_2 _≤...≤ *z*_*n*_, that minimize the deviation $\sum_{i=1}^{n}{\left(z_{i}-y_{i}\right)}^{2}$.

This is the monotone-increasing isotonic regression problem. The monotone-decreasing isotonic regression problem is similar; the only difference is that here *z*_1 _≥ *z*_2 _≥...≥ *z*_*n*_.

The isotonic regression procedure constructs a monotonic number sequence *z*_1_,...,*z*_*n *_(while in linear regression values of *z*_*i *_are linearly expressed in terms of *x*_*i *_: *z*_*i *_= *αx*_*i *_+ *β*).

An algorithm for the solution of isotonic regression problem can be easily constructed. We do not describe it here. We only note that each *z*_*i *_is the mean value of {*y*_*j*_} over a window of variable length: $L_{i}:z_{i}=(\sum_{j=t(i)}^{t(i)+L(i)-1}y_{i})/L_{i}$. For different indices these windows either coincide or do not overlap, and the i-th window contains i.

We also note that the values of *x*_*i *_are not essential in the isotonic regression problem, only the order of values of *y*_*i *_is essential.

To obtain the transfer probabilities using isotonic regression we proceed as follows.

For the evaluation of *p*_1|1 _we consider pairs of similar sequences *π*_*i*_, *π*_*j *_that satisfy the condition *ω *∈ *KW*[*π*_*i*_]. Let *μ*_*ij *_denote the measure of similarity between sequences *π*_*i*_, *π*_*j*_. We put *ξ*_*ij *_= 1 if the word *ω *belongs to the sequence *π*_*j*_, and put *ξ*_*ij *_= 0 if *ω *does not belong to *π*_*j *_(recall that the word *ω *belongs to *π*_*i*_). Then we apply the monotone-increasing isotonic regression procedure to the collection of points (*μ*_*ij*_, *ξ*_*ij*_), where *μ*_*ij *_are in ascending order. The resulting values are the estimates of the transfer probabilities *p*_1|1_(*μ*_*ij*_).

Similarly, applying the monotone-decreasing isotonic regression procedure to the collection of points (*μ*_*ij*_, *ξ*_*ij*_) that correspond to pairs of similar sequences *π*_*i*_, *π*_*j *_for which *ω *does not belong to *π*_*i*_, we obtain values of the transfer probabilities *p*_1|0_(*μ*_*ij*_). As we noted, the probabilities *p*_1|1_, *p*_1|0 _are supposed to satisfy condition (6). Therefore, if it turns out that functions *p*_1|1_(*μ*), *p*_1|0_(*μ*)obtained after the application of isotonic regression procedure do not satisfy this condition for some values of *μ*, then we consider that probabilities *p*_1|1_, *p*_1|0 _are not defined for these values of *μ*. Hence, it is possible that transfer probabilities are defined only for sufficiently large values of *μ*, but not for all *μ*. In particular, it is possible that for some words there are no values of *μ *such that inequality (6) holds. These words are referred to as *degenerate *words.

#### Statistics description

All statistics considered in this paper are based on the likelihood ratio criterion, that is on formula (1) under the assumption of independence of variables *ξ*_*i *_(formula (2)).

Two approaches are used in the definitions of these statistics. One of them can be called a "discrete" approach; the other can be called a "continuous" approach.

The discrete approach is based directly on formulae (1), (2) and the definition of transfer probabilities. It follows from (5) that the following formulae for conditional probabilities hold:

P{*ξ*_*i*_|*ξ*_0 _= 1} = *p*_1|1_(*μ*_*i*_), if *ξ*_*i *_= 1,

P{*ξ*_*i*_|*ξ*_0 _= 1} = *p*_0|1_(*μ*_*i*_) = 1 - *p*_1|1_(*μ*_*i*_), if *ξ*_*i *_= 0,

and hence

(7) $$\text{P}\{\xi_{i}|\xi_{0}=1\}=(1-p_{1|1}(\mu_{i}))\cdot {\left(\frac{p_{1|1}(\mu_{i})}{1-p_{1|1}(\mu_{i}))}\right)}^{\xi_{i}}.$$

Similarly,

(8) $$\text{P}\{\xi_{i}|\xi_{0}=0\}=(1-p_{1|0}(\mu_{i}))\cdot {\left(\frac{p_{1|0}(\mu_{i})}{1-p_{1|0}(\mu_{i})}\right)}^{\xi_{i}}.$$

Relations (7), (8) together with (1), (2) imply the following formula for the logarithm of the likelihood ratio:

(9) $$T^{\left(1\right)}(\xi ;\omega )=T^{\left(1\right)}(\xi )=\ln(\prod_{i=1}^{n}\frac{\text{P}\{\xi_{i}|\xi_{0}=1\}}{\text{P}\{\xi_{i}|\xi_{0}=0\}})=\alpha_{0}+\sum_{i=1}^{n}\alpha_{i}\cdot \xi_{i},$$

where

(10) $$\begin{array}{cc} \alpha_{0}=\sum_{i=1}^{n}\alpha_{0i}, & \alpha_{0i}=\ln\frac{1-p_{1|1}(\mu_{i})}{1-p_{1|0}(\mu_{i})}<0 \end{array}$$

(11) $$\alpha_{i}=\ln\frac{p_{1|1}(\mu_{i})(1-p_{1|0}(\mu_{i}))}{p_{1|0}(\mu_{i})(1-p_{1|1}(\mu_{i}))}>0.$$

One can see that the statistic *T*^(1) ^can be expressed as a linear combination of the *ξ*_*i*_.

As we noted, theoretically the best threshold value for the statistic *T*^(1) ^is equal to 1n1 = 0. However, that is only theoretical. As the assumption of independence of variables *ξ*_*i *_is incorrect and the statistic *T*^(1) ^is only a rough estimate of logarithm of the likelihood ratio, the best threshold value is not necessarily equal to zero (and this threshold does not really equal zero in practice).

Let us put $\eta =\eta (\omega )=\frac{T^{\left(1\right)}-\alpha_{0}}{\sum_{i=1}^{n}\alpha_{i}}$. From the definition of *η *and relations (9)–(11) it follows that

(12) $$\eta =\sum_{i=1}^{n}{\hat{a}}_{i}\xi_{i},$$

where

(13) $${\hat{a}}_{i}=\frac{\ln\frac{p_{1|1}(\mu_{i})(1-p_{1|0}(\mu_{i}))}{p_{1|0}(\mu_{i})(1-p_{1|1}(\mu_{i}))}}{\sum_{i=1}^{n}\ln\frac{p_{1|1}(\mu_{i})(1-p_{1|0}(\mu_{i}))}{p_{1|0}(\mu_{i})(1-p_{1|1}(\mu_{i}))}}>0.$$

We note that

(14) $$\sum_{i=1}^{n}{\hat{a}}_{i}=1.$$

The variable *η*(*ω*) can be treated as a second statistic. Its values lie between 0 and 1. Statistics *T*^(1) ^and *η *are linearly dependent, and hence for a fixed word *ω *these statistics are equivalent. However, the coefficients *α*_0 _and *α*_*i*_(*i *= 1,...,*n*) are different for different words *ω*, so the relation between thresholds, used in the prediction, is different for different words; consequently, the statistics *T*^(1) ^and *η *are not equivalent for the whole totality of words (We will see later that if thresholds are well-chosen, then *η *leads to better results than *T*^(1)^).

The other approach to the definition of statistics, used in the annotation procedure, starts from a linear combination $\eta =\sum_{i=1}^{n}a_{i}\xi_{i}$ of the *ξ*_*i *_as in formula (12). Here coefficients *α*_*i *_are not necessarily given by (13), but should be positive and satisfy the relation (14). This approach uses the assumption that *η *has the normal distribution. Certainly this assumption is not correct (at least because the inequality 0 ≤ *η *≤ 1 always holds). Nevertheless, we use this assumption (and, similarly to the discrete approach, the assumption of independence of variables *ξ*_*i*_(*ω*)). (As the normal distribution is continuous, this approach can be called "continuous").

Thus, we consider a random variable

(15) $$\eta =\sum_{i=1}^{n}a_{i}\xi_{i}$$

and assume that it has a normal distribution. Denote by

$$\begin{array}{c} M_{1}\eta =\text{M}(\eta |\xi_{0}=1),D_{1}\eta =D(\eta |\xi_{0}=1)=\sigma_{1}^{2},\\ M_{0}\eta =\text{M}(\eta |\xi_{0}=0),D_{0}\eta =D(\eta |\xi_{0}=0)=\sigma_{0}^{2} \end{array}$$

the conditional expectations and dispersions of *η *given that *ξ*_0 _= 1 or 0 respectively. We have

*M*_1_*η *= ∑*a*_*i*_M(*ξ*_*i*_|*ξ*_0 _= 1) = ∑*a*_*i*_*p*_1|1_(*μ*_*i*_),   *M*_0_*η *= ∑*a*_*i*_*p*_1|0_(*μ*_*i*_).

Further, the assumption of independence of *ξ*_*i*_(*ω*) implies that

$$\begin{array}{cc} D_{1}\eta =\sigma_{1}^{2}=\sum_{i}a_{i}^{2}\cdot p_{1|1}(\mu_{i})\cdot (1-p_{1|1}(\mu_{i})), & D_{0}\eta =\sigma_{0}^{2}=\sum_{i}a_{i}^{2}\cdot p_{1|0}(\mu_{i})\cdot (1-p_{1|0}(\mu_{i})). \end{array}$$

(We note that it is the only place where the independence of *ξ*_*i *_is used; therefore, the assumption of independence of *ξ*_*i *_is not as essential in this approach as it was in the definition of the statistic *T*^(1)^). It follows from the assumption of a normal distribution that in cases *ξ*_0 _= 1 and 0 the variables

(16) $$\frac{\eta -M_{1}\eta }{\sigma_{1}}\text{ and }\frac{\eta -M_{0}\eta }{\sigma_{0}}\text{ have standard normal distribution N(0,1)}$$

As above, we use the logarithm of the likelihood ratio as a statistic and in addition assume that the ratio of a priori hypothesis probabilities is equal to 1. Let us denote this statistic by *T*^(2)^. Relation (16) implies that

T(2)(λ)=ln⁡P{λ≤η≤λ+dλ|ξ0=1}P{λ≤η≤λ+dλ|ξ0=0}=ln⁡(1/2π⋅σ1)⋅exp⁡{−12⋅((λ−M1η)/σ1)2}(1/2π⋅σ0)⋅exp⁡{12⋅((λ−M0η)/σ0)2}=12⋅{((λ−M0η)/σ0)2−((λ−M1η)/σ1)2}+ln⁡(σ0/σ1).

Another variant of this statistic – statistic ${\hat{T}}^{\left(2\right)}$ – was used in the paper [5]. The statistic ${\hat{T}}^{\left(2\right)}$ is the ratio of type 1 error to type 2 error :

(17) $${\hat{T}}^{\left(2\right)}(\lambda )=\ln\frac{P^{\left(1\right)}(\lambda )}{P^{\left(2\right)}(\lambda )}=\ln\frac{\Phi ((\lambda -M_{1}\eta )/\sigma_{1})}{1-\Phi ((\lambda -M_{0}\eta )/\sigma_{0})},$$

where Φ(*x*) is the cumulative normal distribution function: Φ(x)=12π∫−∞xe−z22dz.

The statistics *η *and ${\hat{T}}^{\left(2\right)}$ are equivalent for an arbitrary fixed word *ω *(although not equivalent for the whole totality of words). At the same time, the statistics *η *and *T*^(2) ^are not necessarily equivalent, as the dependence of *T*^(2) ^on *η *may turn out to be not monotonic. Moreover, it is never monotonic for all values of *η*. However, we are interested only in values 0 ≤ *η *≤ 1, and usually (though not always) the dependence of *T*^(2) ^on *η *is monotonous for these values of *η*, and in this case the statistics *η *and *T*^(2) ^are equivalent (for a fixed word *ω*). Furthermore, even in the case where the dependence is not monotonic, the monotonicity is violated only for the values of *η *close either to 0 or to 1, and the lack of monotonicity can be disregarded.

We still have to discuss the question of the choice of coefficients *a*_*i *_in (15). One of the variants was described above: formula (13) can be applied. Another variant was introduced in [5]. This variant can be described as follows.

As above, we assume that the variate *η *= ∑*a*_*i*_*ξ*_*i *_has a normal distribution. Each set of coefficients *a*_*i *_and each threshold value *λ *have corresponding theoretical frequencies of type 1 and type 2 errors *P*^(1)^(*λ*), *P*^(2)^(*λ*). The idea is to take the set of coefficients {*a*_*i*_} that gives the minimum sum *P*^(1)^(*λ*) + *P*^(2)^(*λ*), where *λ *is the best threshold value for this coefficient set. However, analytically it is very cumbersome, so the following simplification was implemented. For a fixed set of coefficients {*a*_*i*_} we select the threshold value *λ *such that the frequency of type 1 errors is equal to the frequency of type 2 errors: *P*^(1)^(*λ*) = *P*^(2)^(*λ*). Then we find the set of coefficients that minimizes these frequencies *P*^(1)^(*λ*) = *P*^(2)^(*λ*). Here the search of the coefficient set can be reduced to a conditional extremum problem. The *A*^4 ^algorithm uses an iterated procedure for the solution of this problem (see [5]).

In order to optimize the automated annotation procedure, that is to increase the prediction quality, certain modifications of the procedure were introduced. For each statistic (*η*, *T*^(1)^, *T*^(2)^, ${\hat{T}}^{\left(2\right)}$) many variants (up to 36), including the variant that was described above, were considered – each variant corresponds to a certain combination of these modifications. Some variants really turned out to be better than the variants described above.

A simplified scheme of modifications (and thereby of statistic variants) can be described as follows.

1) *What primary local alignments are considered? *In the described variant of statistics only one primary local alignment (the one with the maximum power) was considered. Meanwhile, all constructed local alignments with sufficiently high power can be considered, as it was done in [5]. In this case indices i in (9), (12), (15) correspond not to individual similar sequences, but to primary local alignments of this sequences.

2) *Are the lengths of primary local alignments taken into consideration? *In the described variant lengths of primary local alignments were not taken into consideration, but these lengths can be considered as well. In this case indices i in (9), (12), (15) correspond not to similar sequences or primary local alignments, but to individual positions of these primary local alignments (as in case of FT-type words). In this case the total number of variates *ξ*_*i *_is equal to the overall length of all primary local alignments. For these variants of statistics long local alignments turn out to be more significant than short local alignments with the same power.

3) *How are the coefficients a_i _calculated*? The coefficients *a*_*i *_in (15) can be calculated using different methods – either formula (13) can be used, or an iterative method (described in [5]) can be applied. (For the statistic *T*^(1) ^the coefficients *a*_*i *_are always calculated using formula (13)).

As there are 3 modifications, the total number of basic variants (in the described simplified scheme) equals 2^3 ^= 8 (and for the statistic *T*^(1) ^it equals 4).

So, variants of the statistics *η*, *T*^(1)^, *T*^(2)^, ${\hat{T}}^{\left(2\right)}$ are considered in this paper. Moreover, for the purpose of comparison (as in the paper [5]) the simple statistic

(18) $$q=q(\omega )=\frac{\sum_{i}^{n}\xi_{i}}{n}$$

(the frequency of occurrence of a word *ω *in the collection of similar sequences) is also considered.

**Figure 1:**
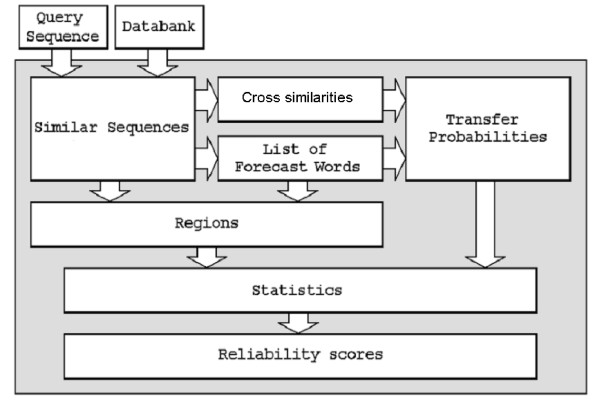
A^4 ^algorithm scheme.

Finally we quote a scheme of the *A*^4 ^algorithm in Figure 1 (this scheme is essentially taken from [5]). A short description of each stage can be found in [5]. Here we only note that the most time-consuming stage is the first one – the generation of a collection of similar sequences. We also note that in the current investigation regions were not determined, and the prediction was performed for the whole query sequence.

### Testing results

#### Testing scheme

A collection of 518 sequences, randomly selected from SWISS-PROT databank, was generated. Note that only initially annotated sequences (i.e., sequences whose description fields were not obtained by the extension from similar sequences) were selected. The prediction was performed for KW-type words. All selected sequences were divided into two groups. The first group contained 210 sequences. This group was used during the "learning stage": the procedure was applied to all sequences from the group, and values of procedure parameters (including optimal threshold values) that minimize the total number of errors for the first group were selected. The remaining 308 sequences were used for the "main testing" that was performed using parameter values obtained on the basis of "learning" results. A list of all these 308 sequences is given in Additional file 1.

The total number of KW-type words in the description fields of 308 selected sequences, used as query sequences, was equal to 1176 (the positive prediction was preferable for these words). As it was noted, a collection of similar sequences was generated for each query sequence; the number of sequences in these collections equaled 100. Then the list of all KW-type words from description fields of similar sequences was formed. The majority of these words belonged only to one similar sequence. All such words are degenerate, transfer probabilities are not defined for them. At the same time nearly all these words did not belong to the query sequence. Hence it was sensible to perform prediction only for words that belonged to at least two similar sequences. For all these words transfer probabilities were evaluated, non-degenerate words (i.e., words with defined transfer probabilities) were determined, and the prediction was performed (for both degenerate and non-degenerate words).

Table 1 shows certain characteristics of sequences used for the testing.

**Table 1:** Certain characteristics of sequences used for the testing

Total number of query sequences, i.e., sequences used during the main testing	308
Total number of words that belong to query sequences (*n*_*q*_)	1176
Including: non-degenerate words	824
degenerate words	352
Total number of words for which the prediction was performed (*n*_*all*_)	9236
Including: non-degenerate words	7207
degenerate words	2029

The average number of words considered per query sequence equals 30 and the entire range was from 3 to 67.

#### Final results

Testing results are presented in Table 2.

**Table 2:** Testing results for different basic variants of the statistics.

Statistic	*N*^1^	*N*^2^	*N*^*all*^	*P*^(1) ^+ *P*^(2)^	*P*^(1) ^+ *P*^(+)^
*η*[1,1]	***118***	***102***	***220***	***0.113***	***0.188***
*η*[1,0]	130	104	234	0.123	0.201
*η*[0,1]	136	106	242	0.129	0.208
*η*[0,0]	164	103	267	0.152	0.232
*T*^(1)^[1,0]	***213***	***83***	***296***	***0.191***	***0.260***
*T*^(2)^[1,1]	***172***	***124***	***296***	***0.162***	***0.256***
*T*^(2)^[0,1]	216	103	319	0.196	0.281
*T*^(2)^[1,0]	180	141	321	0.171	0.277
*T*^(1)^[0,0]	200	124	324	0.185	0.283
${\hat{T}}^{\left(2\right)}$[0,1]	***189***	***136***	***325***	***0.178***	***0.282***
${\hat{T}}^{\left(2\right)}$[0,0]	234	111	345	0.213	0.304
${\hat{T}}^{\left(2\right)}$[0,0]	205	148	353	0.193	0.307
${\hat{T}}^{\left(2\right)}$[1,1]	142	217	359	0.148	0.294
${\hat{T}}^{\left(2\right)}$[1,0]	164	224	388	0.167	0.321
*q*	***393***	***158***	***551***	***0.354***	***0.502***
*S*_*And*_	***332***	***174***	***506***	***0.304***	***0.489***

The table shows the testing results of the different statistics for *n*_*all *_= 9236 'predictions'. In *n*_*q *_= 1176 cases the "predicted word" belonged to the sequence, in *n*_*all *_- *n*_*q *_= 8060 it did not. *N*^1 ^denotes the number of false negatives, *N*^2 ^is the number of false positives. *N*^*all *^= *N*^1 ^+ *N*^2^, *P*^(1) ^= *N*^1^/*n*_*q*_, *P*^(2) ^= *N*^2^/(*n*_*all *_- *n*_*q*_) and *P*^(+) ^= *N*^2^/*N*^2 ^+ (*n*_*q *_- *N*^1^)). See the main text for the description of the variants.

The table contains testing results for the basic statistics *η*, *T*^(1)^, T^(2)^, ${\hat{T}}^{\left(2\right)}$ as well as for the statistics *q *and *S*_*And*_, included into the table for the purpose of comparison (See the Discussion section). Each line of the table corresponds to a certain variant of studied statistics. The first column shows which statistic and which variant of this statistic corresponds to a line. Variants are given in square brackets: the first number equals 0 if lengths of primary local alignments are not considered, and 1 otherwise; the second number equals 0 if the coefficients *a*_*i *_are calculated using formula (13), and 1 if the coefficients *a*_*i *_are calculated using the iterative procedure (for the statistic *T*^(1) ^the second number is always 0). The second column contains the number *N*^1 ^of type 1 errors (i.e., number of cases when the prediction for a word that belongs to description fields of a query sequence is negative). The third column contains the number *N*^2 ^of type 2 errors (i.e., number of cases when the prediction for a word that does not belong to description fields of a query sequence is positive). The forth column contains the total number of errors *N*^*all *^= *N*^1 ^+ *N*^2^. The next columns contain sums *P*^(1) ^+ *P*^(2) ^and *P*^(1) ^+ *P*^(+)^, where *P*^(1) ^is the proportion of type 1 errors, *P*^(2) ^is the proportion of type 2 errors, and *P*^(+) ^is the ratio of false positive predictions to the total number of predictions: $P^{\left(1\right)}=\frac{N^{1}}{n_{q}}$, $P^{\left(2\right)}=\frac{N^{2}}{n_{all}-n_{q}}$, $P^{\left(+\right)}=\frac{N^{2}}{n_{+}}$ (*n*_*all *_is the total number of words for which the prediction is performed, i.e., total number of KW-type words in description fields of all sequences, similar to at least one query sequence, *n*_*q *_is the number of words (from the list of these *n*_*all *_words) that belong to query sequences, *n*_+ _is the total number of words for which the prediction is positive; here *n*_*all *_= 9236, *n*_*q *_= 1176, *n*_*all *_- *n*_*q *_= 8060; the value of *n*_+ _of depends on the threshold evaluated for the given version of statistic at the "learning stage"). Lines are ordered according to the prediction quality: higher lines correspond to statistic variants with lower total number of errors. For a fixed statistic, lines that correspond to the best variant of this statistic (i.e., for the variant that leads to the lowest total number of errors) are marked. Recall that the statistic ${\hat{T}}^{\left(2\right)}$[1,0] is exactly the statistic that was considered in [5] (note that it is the best variant for the statistic ${\hat{T}}^{\left(2\right)}$).

The results could also have been presented as a confusion table as laid out in Table 3, but doing so for all variants would take a lot of space.

**Table 3:** Lay out of the confusion table for the results of Table 2.

	word "predicted"	word "not predicted"	total
word present	1176-*N*^1^	*N *^1^	*n*_*q *_= 1176
word absent	*N*^2^	8060-*N*^2^	*n*_*all *_- *n*_*q *_= 8060
total	1176-*N*^1^+ *N*^2^	*N*^1 ^+ 8060-*N*^2^	*n*_*all *_= 9236

The testing results showed that the first modification (consideration of all primary local alignments or only one primary local alignment with the maximum power) did not significantly affect the prediction quality, so results are only presented for one case (all local alignments are considered, similarly to [5]).

Note that the results presented in the Table correspond to the whole totality of words including degenerate words (though statistics *η*, *T*^(1)^, *T*^(2)^, ${\hat{T}}^{\left(2\right)}$, *T*^(*nik*) ^were evaluated only for non-degenerate words, for degenerate words the prediction was performed on the basis of the statistic *q*, i.e., on the basis of word frequency). In particular, the total number of errors includes errors for degenerate words. It is worth noticing that the total number of errors for degenerate words in case of the best choice of threshold *q*_0 _for the frequency *q*(*ω*) turned out to be surprisingly small: only 13 (whereas the number of errors for the whole totality of words was 220); these errors included twelve type 1 errors and one type 2 error (recall that the prediction was performed for 2029 degenerate words, and the prediction was incorrect only in 13 cases). Such a small number of errors can seemingly be explained by the fact that for degenerate words the frequency *q*(*ω*) is nearly always close either to 1 or to 0, otherwise in almost all cases transfer probabilities *p*_1|1_, *p*_1|0 _can be defined for at least some values of similarity measure *μ *and hence a word *ω *turns out to be non-degenerate.

We also note that along with type 1 and type 2 errors other errors can occur, as it is possible that some words from description fields of a query sequence do not belong to description fields of similar sequences and hence the prediction is not performed for these words at all. However, we used a large number of similar sequences (100) for the prediction, so such errors were extremely rare (only two words for the whole test set of 518 sequences, whereas the number of words for which the prediction was performed equaled 9236). Hence, in our case these errors can be disregarded.

Statistical analysis showed that the precision of the evaluation of *N*^1^, *N*^2^, *N*^*all *^is reasonable: it can be checked that the relative precision of *N*^*all *^(the standard error of ln(*N*^*all*^)) is in the order of 7–10% which is in line with the relative precision of a Poisson random variable that is given by $1/\sqrt{N^{all}}$. However, since the "methods" (the statistics and their variants) are compared on the same sequences, the standard error for the comparison between methods is much smaller due to the high correlation of results for the same sequence and is in the order of 2–4%. This implies that "methods" for which *N*^*all *^differ by more than 10% can considered to be significantly different.

This shows that size of the experiment with 210 randomly selected sequences in the learning stage and 308 sequences in the testing stage is large enough to obtain valid statements about the accuracy of the proposed methodology and allows statistical comparisons of different statistics and variants.

When the set of tested sequences is fixed, the total number of errors *N*^*all *^is an objective characteristic of prediction quality, and the optimal threshold values (selected during the "learning stage") provide exactly the minimum of the total number of errors. However, if different testing results (based on different sets of query sequences) are compared, then absolute numbers of errors can not be treated as a procedure quality measure, and relative quantities (proportions) should be considered instead of absolute quantities. Usually a sum of the proportion of type 1 errors and the proportion of type 2 errors *P*^(1) ^+ *P*^(2) ^is used as a quality measure; values of this sum are presented in the fifth column of Table 2. However, in our situation the number of words *n*_*all *_- *n*_*q *_that do not belong to query sequences is much larger than *n*_*q*_, and for the majority of these words it is obvious that they do not belong to a query sequence. Consequently, in case of optimal parameter values the proportion of type 2 errors *P*^(2) ^is small, it is considerably smaller than *P*^(1)^. Thus the quantity *P*^(1) ^+ *P*^(2) ^is not representative in our case (see [5]). The ratio *P*^(+) ^of the number of wrong positive predictions to the total number of positive predictions is more representative than *P*^(2)^. Hence, it is natural to measure procedure quality by the sum *P*^(1) ^+ *P*^(+)^. Exactly this procedure quality measure was used in [5].

**Figure 2:**
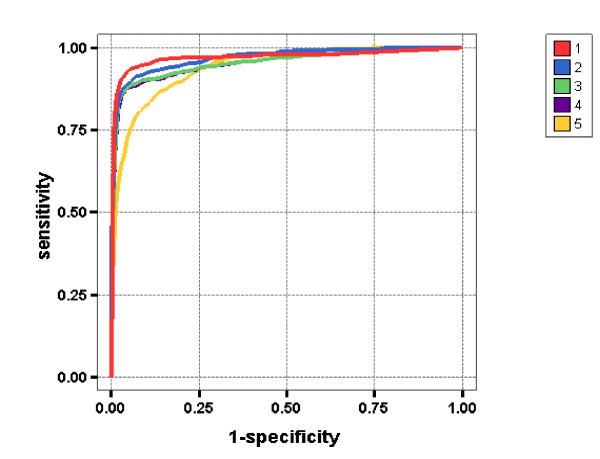
**ROC curves for the best variants of the statistics**. The figure shows the ROC curves (1–5) for the best variants of the statistics (i.e., for variants marked in Table 2). Curve 1 corresponds to *η*[1,1]; curve 2 corresponds to *T*^(1)^[1,0]; curve 3 corresponds to *T*^(2)^[1,1]; curve 4 corresponds to ${\hat{T}}^{\left(2\right)}$[0,1]; curve 5 corresponds to q.

In medical decision making (diagnostic testing) the terms sensitivity (*sens *= 1 - *P*^(1)^) and specificity (*spec *= 1 - *P*^(2)^) are frequently used to quantify the accuracy of a procedure, while the quantity 1 - *P*^(+) ^is known as the positive predictive value. Alternative terminology is discussed in [12]. The ROC curve plotting *sens *against 1 - *spec *is a popular way of showing the overall performance of a diagnostic test without specification of a cut off value. It is also used and discussed in [12], but with different labels for the axes. Figure 2 contains ROC curves for the best variants of the statistics (i.e., for variants that were marked in Table 2) applied to the non-degenerate words.

## Discussion

### Overall comparison of the statistics *T*^(1)^, *η*, *T*^(2)^, ${\hat{T}}^{\left(2\right)}$

Mean values of *N*^*all *^(where averaging is performed over all variants presented in Table 2) for different statistics are the following: 241 for *η*, 310 for *T*^(1)^, 320 for *T*^(2)^, and 356 for ${\hat{T}}^{\left(2\right)}$ (recall that differences in the order of 10% can be considered to be significant). These numbers show that the statistics can clearly be ordered with respect to the prediction quality: the best statistic is *η*, then comes *T*^(1)^, then *T*^(2)^, and finally ${\hat{T}}^{\left(2\right)}$. For the comparison of the statistics *η*, *T*^(1)^, as well as for the comparison of statistics the *T*^(2)^, ${\hat{T}}^{\left(2\right)}$ this conclusion is obvious. For the comparison of the statistics *T*^(1)^, *T*^(2) ^it seems to be less obvious, but the consideration of variants in which the calculation of coefficients *a*_*i *_is performed using formula (13) (i.e., variants [0,0] and [1,0]; recall that the statistic *T*^(1) ^has only such variants) clearly shows that the statistic *T*^(1) ^is considerably better than *T*^(2)^. The same conclusion can be drawn from the comparison of ROC curves presented in Fig. 2.

Testing results show that the prediction quality for the best variant of the statistic *η *(see the first line of Table 2) is much higher (higher by 50%) in comparison with results of [5] (see the line of Table 2 that corresponds to ${\hat{T}}^{\left(2\right)}$[0,1]). That is the most striking finding of the current paper.

It is interesting to see that the statistic *η *turned out to be better than *T*^(1)^: the latter would be expected to be better as it equals the logarithm of the likelihood ratio (in reality, it does not equal this logarithm because the *ξ*_*i *_are not independent). We suppose that this fact can be explained as follows. Recall that *T*^(1) ^and *η *are equivalent for an arbitrary fixed word *ω*. These statistics turn out to be not equivalent only if they are compared on the whole totality of words. It is worth noting that the variation of the values of the statistic *T*^(1) ^(i.e., the difference between values of *T*^(1) ^in cases *ξ*_1 _=....= *ξ*_*n *_= 0 and *ξ*_1 _=....= *ξ*_*n *_= 1) significantly depends on a word *ω*: for some words these values vary from -700 to 700, for some other from -0.01 to 0.01. In principle, the optimal threshold value is different for different words. As the ranges of *T*^(1) ^values essentially vary, then it is probable that optimal thresholds also essentially vary. The optimal threshold for the whole totality of words is a certain mean value of thresholds over individual words *ω*. Since the optimal thresholds are significantly different for different words, the quality of the mean is low: for certain words (e.g., words with small variation of *T*^(1)^) this mean is completely unrepresentative. In the same time, the range of values of *η *is the same for all words (these values lie between 0 and 1). Consequently, the difference between optimal thresholds for individual words is probably not so significant, and the mean gives better quality for individual words in comparison with *T*^(1)^.

From the point of view of prediction quality the statistic *T*^(2) ^turned out to be worse than *T*^(1)^. It is not surprising, because during the derivation of the formula for *T*^(2) ^along with the incorrect assumption of independence of *ξ*_*i *_we also made the incorrect assumption of the normal distribution of *η*. This consideration is applicable to ${\hat{T}}^{\left(2\right)}$ as well. Since the cut-off points for these statistics are determined empirically in the test set and validated in the training set, the violation of the normality assumption does not invalidate the procedures as such, but might affect their performance.

### Effect of procedure modifications

The next issue is the dependence of prediction quality on procedure modifications. One can see that for a fixed statistic the prediction quality significantly depends on the choice of the variant. Thus, the introduction of modifications turned out to be effective.

It is interesting that all statistics for which coefficients *a*_*j *_can be calculated in different ways (these are the statistics *η*, *T*^(2) ^${\hat{T}}^{\left(2\right)}$) prediction quality was better in case when these coefficients were calculated using the iterative procedure.

At the same time the dependence of prediction quality on the modification related to the consideration of lengths of primary local alignments is more intricate: for the statistic *η*, and to a lesser extent for the statistics *T*^(1)^, *T*^(2)^, the results are better if lengths of primary local alignments are considered, but for the statistic ${\hat{T}}^{\left(2\right)}$ results are better when lengths of primary local alignments are not considered. Currently reasons of this fact are not clear for us.

As it was noted, the dependence of results on modifications dealing with the number of considered primary local alignments for similar sequences is not essential. (However, we note that for nearly all variants that were described above as well as variants that were not described, prediction quality is better in case when all primary local alignments are considered).

### Comparison with other procedures

For the purpose of comparison we compared our findings with the simple statistic *q*(*ω*) (the frequency of word occurrence in the list of similar sequences, see (18)) was also considered, using a threshold of *q *= 0.422. As expected, *q*(*ω*) gave essentially worse results in comparison with *T*^(1)^, *η*, *T*^(2)^, ${\hat{T}}^{\left(2\right)}$ (see Table 2).

Furthermore, we compared our results with the results, which predicts all of the words for which there is among similar sequences at least one sequence with a power above a certain threshold. Such an approach was used by Andrade M. et al. [2]. This is the statistics *S*_*And *_as already mentioned in Table 2. It is defined as *S*_*And*_(*ω*) = max *μ*_*j*_, where *μ*_*j *_is the measure of similarity between sequences *π*_0_, *π*_*j*_, and the maximum is taken on the j for which *ξ*_*j*_(*ω*) = 1 (i.e. the word belongs to these sequences). In our application similarity is measured by the power value (see [9,10]) as used in the other statistics in this paper and mentioned before, while [2] used the E-value. That makse the cut-offs hard to compare. The results for these statistics are given in the same Table 2. The results were better than in the statistics *q*(*ω*), but worse than in any of our statistics.

Surprisingly, it turned out that the prediction quality is better when only some similar sequences (e.g., 10 from 100) are used for the evaluation of a statistic (but not for the evaluation of transfer probabilities!). It means that a large part of similar sequences only leads to an increase of "noise". (See [13] for details.)

We would also like to mention the work [14] on FunCat categories containing a set of about 7,500 well annotated proteins and providing a benchmarking for different methods of automated annotation. It would be interesting to apply our procedure to this database, but that has not been realized yet.

We did not attempt at this stage to apply our approach to the GO annotation. It would be interesting further research to switch to GO data and to compare our approach with other approaches in the literature.

There is a similarity with the approach of Kajan et al. [15]. They also base their procedures on the likelihood ratio, but use approximations based on maximal similarity (or minimal distance), while we attempt to estimate the likelihood ratio from pairwise comparisons within the set of similar sequences.

There is also an interesting relation with the GOPET tool presented in [16] based on earlier work by the same authors [17]. These authors use a number of characteristics of the set of found similar sequences for term (word), such as maximal e-value, frequency of the term etc., as a coordinates of decision making space. They use more features while we concentrate on the similarity. That might be an advantage for their method. On the other hand we try to use the information from all similar sequences in an optimal way relying on statistical decision theory by means of the use of transfer probabilities and the concept of likelihood ratio. It is an interesting topic of further research to compare the two methods.

So far we only predicted the presence of a key word. It is quite a challenge to obtain a true prediction of function.

## Conclusion

The main conclusion of the paper is that the introduction of the concept of likelihood ratio coming from statistical decision theory is very helpful in the development of automated annotation procedures. We obtained a substantial improvement when compared with our previous results.

We are sure that there is room for further improvement. Issues for further research are the size of the set of similar sequences and the combination of different statistics into a "super-predictor".

## Authors' contributions

A.M. Leontovich and H.C. van Houwelingen developed the approach. A.M. Leontovich developed the methodology and the algorithms in cooperation with K.Y. Tokmachev and H.C. van Houwelingen. K.Y. Tokmachev carried out all computations.

## Supplementary Material

Additional file 1 Testing sequences. The file contains the list of the 308 sequences used in the testing phase. Click here for file

## References

[B1] Fleischmann W, Moller S, Gateau A, Apweiler R (1999). A novel method for automatic functional annotation of proteins. Bioinformatics.

[B2] Andrade MA, Brown NP, Leroy C, Hoersch S, de Daruvar A, Reich C, Franchini A, Tamames J, Valencia A, Ouzounis C, Sander C (1999). Automated genome sequence analysis and annotation. Bioinformatics.

[B3] Kretschmann E, Fleischmann W, Apweiler R (2001). Automatic rule generation for protein annotation with the C4.5 data mining algorithm applied on SWISS-PROT. Bioinformatics.

[B4] Hegyi H, Gerstein M (2001). Annotation transfer for genomics: Measuring functional divergence in multi-domain proteins. Genome Research.

[B5] Leontovich AM, Brodsky LI, Drachev VA, Nikolaev VK (2002). Adaptive algorithm of automated annotation. Bioinformatics.

[B6] Cox DR, Hinkley DV (1974). Theoretical Statistics.

[B7] Durbin R, Eddy S, Krogh A, Mitchison G (1998). Biological sequence analysis Probabilistic models of proteins and nuclear acids.

[B8] Altschul SF, Gish W, Miller W, Myers EW, Lipman DJ (1990). Basic local alignment search tool. Journal of Molecular Biology.

[B9] Leontovich AM, Brodsky LI, Gorbalenya AE (1993). Construction of the full local similarity map for 2 biopolymers. Biosystems.

[B10] Altschul SF, Erickson BW (1986). Optimal sequence alignment using affine gap costs. Bulletin of Mathematical Biology.

[B11] Barlow RE, Bartholomew JM, Bremner JM, Brunk HD (1972). Statistical Inference Under Order Restrictions.

[B12] Baldi P, Brunak S, Chauvin Y, Andersen CAF, Nielsen H (2000). Assessing the accuracy of prediction algorithms for classification: an overview. Bioinformatics.

[B13] Leontovich AM, Tokmachev KY (2006). Methods for improving the quality of prediction in the process of automatic annotating A(4). Biofizika.

[B14] Ruepp A, Zollner A, Maier D, Albermann K, Hani J, Mokrejs M, Tetko I, Guldener U, Mannhaupt G, Munsterkotter M, Mewes HW (2004). The FunCat, a functional annotation scheme for systematic classification of proteins from whole genomes. Nucleic Acids Res.

[B15] Kajan L, Kertesz-Frarkas A, Franklin D, Ivoanova N, Kocsor A, Pongor S (2006). Application of a simple likelihood ratio approximant to protein classification. Bioinformatics.

[B16] Vinayagam A, del Val C, Schubert F, Eils R, Glatting KH, Suhai S, König R (2006). GOPET: A tool for automated predictions of Gene Ontology terms. BMC Bioinformatics.

[B17] Vinayagam A, König R, Moormann J, Schubert F, Eils R, Glatting KH, Suhai S (2004). Applying Support Vector Machines for Gene ontology based gene function prediction. BMC Bioinformatics.

